# Supplementary material for: Urban-Rural Gaps in Breastfeeding Practices: Evidence From Lao People’s Democratic Republic
Source: Int J Public Health. 2021 Sep 9;66:1604062. doi: 10.3389/ijph.2021.1604062 (PMC8458572; doi:10.3389/ijph.2021.1604062)
Supplement: Supplementary file 2 [file DataSheet1.docx]

| **Appendix- Table 1. Complete Case Analysis: Predictors of non-Compliance with World Health Organization Recommendations to Exclusively Breastfeed during the first 6 months of life, Lao Social Indicator Survey II, Lao People's Democratic Republic, 2017.** | | | | | |
| --- | --- | --- | --- | --- | --- |
|  | **Bivariate** | **Model 1**  Adjusted for Maternal Demographic Factors  (N=1233) | **Model 2**  Adjusted for Model 1  +  Child Factors  (N=1233) | **Model 3**  Adjusted for Model 2  +  Socioeconomic Status  (N=1233) | **Fully Adjusted**  (N=1139) |
| **Predictor** |  | **OR (95% CI)** | | | |
| **Residence**  Large-Urban -Vientiane Capital  Small- Urban -Other Province  Rural | 3.65 (1.48, 9.00)***  0.95 (0.73, 1.25)  Reference | 3.39 (1.36, 8.44)***  0.93 (0.71, 1.23)  Reference | 4.64 (1.77, 12.19)**  1.04 (0.76, 1.41)  Reference | 2.75 (0.99, 7.66)  0.76 (0.54, 1.09)  Reference | 3.98 (1.17, 13.57)*  0.84 (0.58, 1.22)  Reference |
| **Marital Status**  (married vs not married) | 0.73 (0.40, 1.33) | 0.75 (0.41, 1.38) | 0.65 (0.33, 1.27) | 0.68 (0.34, 1.36) | 0.90 (0.41, 2.00) |
| **Maternal Age**  <20 years  20-25 years  26-29 years  30-35 years  >35 years | 0.74 (0.45, 1.21)  072 (0.46, 1.12)  0.87 (0.54, 1.40)  0.80 (0.49, 1.30)  Reference | 0.73 (0.45, 1.21)  0.72 (0.46, 1.12)  0.76 (0.43, 1.37)  0.78 (0.47, 1.27)  Reference | 0.66 (0.38, 1.15)  0.57 (0.35, 0.94)*  0.70 (0.41, 1.18)  0.73 (0.42, 1.25)  Reference | 0.77 (0.43, 1.36)  0.63 (0.38, 1.06)  0.76 (0.45, 1.31)  0.76 (0.44, 1.32)  Reference | 0.76 (0.41, 1.41)  0.67 (0.39, 1.17)  0.78 (0.44, 1.38)  0.84 (0.46, 1.51)  Reference |
| **Child Sex**  (Male vs Female) | 0.81 (0.65, 1.01) | -- | 0.79 (0.62, 1.02) | 0.79 (0.61, 1.02) | 0.79 (0.60, 1.03) |
| **Children’s Age**  0-2 months  3-4 months  5-6 months | Reference  2.52 (1.91, 3.32)***  12.28 (8.72, 17.30)*** | -- | Reference  2.64 (1.99, 3.51)***  13.00 (9.17, 18.43)*** | Reference  2.74 (2.06, 3.66)***  13.76 (9.65, 19.60)*** | Reference  3.05 (2.25, 4.14)***  14.20 (10.47, 22.07)*** |
| **Maternal Education**  No Schooling  Primary  Lower Secondary  Upper Secondary  Post Secondary or Higher | Reference  0.92 (0.68, 1.25)  1.03 (0.73, 1.46)  0.97 (0.63, 1.50)  1.36 (0.88, 2.09) | -- | -- | Reference  0.88 (0.61, 1.27)  0.82 (0.53, 1.29)  0.75 (0.43, 1.32)  0.77 (0.43, 1.37) | Reference  0.75 (0.51, 1.12)  0.73 (0.45, 1.19)  0.75 (0.41, 1.38)  0.66 (0.35, 1.25) |
| **Wealth Index**  Poorest  Second  Middle  Fourth  Richest | Reference  1.18 (0.87, 1.60)  1.21 (0.87, 1.67)  1.70 (1.20, 2.43)**  1.89 (1.27, 2.80)** | -- | -- | Reference  1.24 (0.87, 1.78)  1.47 (0.98, 2.19)  2.44 (1.54, 3.86)**  2.81 (1.60, 4.92)** | Reference  1.21 (0.82, 1.78)  1.32 (0.85, 2.05)  2.19 (1.32, 3.63)**  2.42 (1.31, 4.46)** |
| **Attitude that domestic violence is not acceptable**  (no vs yes) | 0.87 (0.69, 1.11) | -- | -- | -- | 0.93 (0.70, 1.23) |
| **Prenatal Care**  (no vs yes) | 0.94 (0.70, 1.26) | -- | -- | -- | 0.97 (0.63, 1.50) |
| **Baby put directly on bare skin of mothers chest after birth**  (no vs yes) | 0.93 (0.74, 1.18) | -- | -- | -- | 1.33 (0.95, 1.85) |
| **Healthcare provider observed child’s breastfeeding within 2 days after birth**  (no vs yes) | 0.62 (0.42, 0.90)* | -- | -- | -- | 0.89 (0.46, 1.73) |
| **Healthcare provider counseled on breastfeeding within 2 days after birth**  (no vs yes) | 0.72 (0.51, 1.00) | -- | -- | -- | 0.85 (0.46, 1.57) |
| **Place of Birth**  Public Sector  Private Medical Sector  Home | Reference  1.26 (0.21, 7.60)  0.77 (0.61, 0.98)* | -- | -- | -- | Reference  0.70 (0.07, 6.82)  0.76 (0.52, 1.11) |

**p*-value <0.05 ***p*-value <0.01 ****p*-value <0.001; OR= odds ratio; CI= confidence interval

**Appendix- Table 2. Complete Case Analysis: Predictors of Non-Compliance with World Health Organization Recommendations to Complementary Breastfeed between 6 and 23 months, Lao Social Indicator Survey II, Lao People's Democratic Republic, 2017.**

|  | **Bivariate** | **Model 1**  Adjusted for Maternal Demographic Factors  (N=3029) | **Model 2**  Adjusted for Model 1  +  Child Factors  (N=3029) | **Model 3**  Adjusted for Model 2  +  Socioeconomic Status  (N=3026) | **Fully Adjusted**  (N=2752) |
| --- | --- | --- | --- | --- | --- |
| **Predictor** | | **OR (95% CI)** | | | |
| **Residence**  Large-Urban -Vientiane Capital  Small- Urban -Other Province  Rural | 4.23 (2.82, 6.34)***  2.04 (1.73, 2.42)***  Reference | 3.98 (2.64, 6.02)***  2.03 (1.71, 2.41)***  Reference | 6.06 (3.73, 9.85)***  2.46 (2.01, 3.02)***  Reference | 1.90 (1.12, 3.24)*  1.15 (0.90, 1.46)  Reference | 2.02 (1.14, 3.56)*  1.19 (0.92, 1.54)  Reference |
| **Marital Status**  (married vs not married) | 1.06 (0.70, 1.60) | 0.98 (0.65, 1.50) | 0.99 (0.61, 1.62) | 0.97 (0.59, 1.62) | 1.19 (0.68, 2.07) |
| **Maternal Age**  <20 years  20-25 years  26-29 years  30-35 years  >35 years | 0.54 (0.39, 0.76)**  0.83 (0.64, 1.07)  0.94 (0.71, 1.23)  0.91 (0.69, 1.20)  Reference | 0.62 (0.45, 0.87)**  0.92 (0.70, 1.20)  0.91 (0.69, 1.21)  0.89 (0.67, 1.18)  Reference | 0.67 (0.46, 0.99)*  0.91 (0.67, 1.24)  0.93 (0.68, 1.29)  0.87 (0.63, 1.20)  Reference | 0.75 (0.50, 1.13)  0.86 (0.62, 1.20)  0.76 (0.53, 1.07)  0.70 (0.50, 1.00)  Reference | 1.06 (0.67, 1.69)  1.11 (0.76, 1.63)  0.98 (0.65, 1.46)  0.88 (0.59, 1.32)  Reference |
| **Child Sex**  (Female vs Male) | 1.04 (0.90, 1.20) | -- | 1.01 (0.85, 1.20) | 0.99 (0.83, 1.19) | 1.02 (0.84, 1.24) |
| **Child Age At Interview**  7-8 Months  9-10 Months  11-12 Months  13-14 Months  15-16 Months  17-18 Months  19-20 Months  21-22 Months  23-24 Months | Reference  0.98 (0.64, 1.49)  1.64 (1.11, 2.42)*  2.60 (1.79, 3.79)***  5.70 (3.97, 8.19)***  10.25 (7.13, 14.74)***  12.82 (8.89, 18.48)***  15.95 (11.03, 23.05)***  24.78 (15.46, 39.69)*** | -- | Reference  1.06 (0.68, 1.65)  1.73 (1.14, 2.62)*  2.80 (1.87, 4.18)***  6.88 (4.67, 10.13)***  12.19 (8.27, 18.00)***  15.13 (10.25, 22.32)***  19.30 (13.04, 28.56)***  31.12 (18.95, 51.09)*** | Reference  1.05 (0.67, 1.66)  1.76 (1.15, 2.70)**  3.12 (2.05, 4.75)***  8.69 (5.78, 13.07)***  14.27 (9.49, 21.48)***  19.08 (12.67, 28.73)***  24.76 (16.38, 37.41)***  45.04 (26.78, 75.75)*** | Reference  1.02 (0.62, 1.67)  1.73 (1.09, 2.75)*  3.42 (2.19, 5.34)**  9.29 (6.00, 14.36)***  14.94 )9.66, 23.12)***  20.81 (13.45, 32.20)***  27.69 (17.80, 43.06)***  60.10 (34.06, 106.03)*** |
| **Maternal Education**  No Schooling  Primary  Lower Secondary  Upper Secondary  Post Secondary or Higher | Reference  1.18 (0.87, 1.60)  1.21 (0.87, 1.67)  1.70 (1.20, 2.43)**  1.89 (1.27, 2.80)** | -- | -- | Reference  1.65 (1.26, 2.16)**  1.55 (1.14, 2.12)**  1.55 (1.01, 2.36)*  3.01 (2.00, 4.55)*** | Reference  1.64 (1.21, 2.21)**  1.46 (1.04, 2.07)*  1.42 (0.90, 2.24)  3.11 (1.99, 4.87)** |
| **Wealth Index**  Poorest  Second  Middle  Fourth  Richest | Reference  1.55 (1.24, 1.93)***  2.64 (2.11, 3.31)***  4.21 (3.32, 5.35)***  5.64 (4.38, 7.27)*** | -- | -- | Reference  1.61 (1.24, 2.09)**  2.86 (2.15, 3.81)***  4.73 (3.42, 6.54)***  5.32 (3.56, 7.94)*** | Reference  1.50 (1.12, 2.00)*  2.68 (1.95, 3.70)***  4.29 (2.97, 6.20)***  5.12 (3.27, 8.01)*** |
| **Attitude that domestic violence is not acceptable**  (no vs yes) | 1.10 (0.84, 1.29) | -- | -- | -- | 1.12 (0.91, 1.37) |
| **Prenatal Care**  (no vs yes) | 0.46 (0.38, 0.57)*** | -- | -- | -- | 0.59 (0.43, 0.80)** |
| **Baby put directly on bare skin of mothers chest after birth**  (no vs yes) | 0.74 (0.64, 0.87)** | -- | -- | -- | 1.02 (0.81, 1.29) |
| **Healthcare provider observed child’s breastfeeding within 2 days after birth**  (no vs yes) | 0.63 (0.50, 0.80)** | -- | -- | -- | 0.95 (0.58, 1.55) |
| **Healthcare provider counseled on breastfeeding within 2 days after birth**  (no vs yes) | 0.64 (0.51, 0.79)*** | -- | -- | -- | 1.04 (0.66, 1.63) |
| **Place of Birth**  Public Sector  Private Medical Sector  Home | Reference  1.84 (1.04, 3.24)*  0.58 (0.49, 0.68)*** |  |  |  | Reference  1.05 (0.50, 2.20)  1.26 (0.97, 1.64) |
|  |  |  |  |  |  |

**p*-value <0.05 ***p*-value <0.01 ****p*-value <0.001; OR= odds ratio; CI= confidence interval

|  |  |  |  |
| --- | --- | --- | --- |

|  |  |  |  |
| --- | --- | --- | --- |
